# Ionizing Radiation: Friend or Foe of the Origins of Life?

**DOI:** 10.1007/s11084-012-9314-1

**Published:** 2012-10-19

**Authors:** Z. P. Zagórski, E. M. Kornacka

**Affiliations:** Centre for Radiation Research, Institute of Nuclear Chemistry and Technology, PL-03-195 Warsaw, Poland

**Keywords:** Origins of life, Ionizing radiation, Prebiotic reactions, Radiation induced reactions, Evolution

Ionizing radiation is defined as electromagnetic or corpuscular radiation, of energy of quanta resp. particles, which are able to detach an electron from any atom or molecule, as an object of interaction. The act of ionization creates reactive species like ion-radicals and free radicals, which start sequences of chemical reactions even of high activation energies. Similar effects can be started by another energetic interactions of existing energy, close to ionizing radiation, e.g. by electrical discharges in gases like an atmosphere of a planet. Lightning, not strictly speaking ionizing radiations but rather a source of high energy chemistry was very early responsible for more concentrated deposition of energy than by ionizing radiation, calculating the amount of energy per unit of volume. Therefore it was easier to notice the connection to the beginnings of life, as Miller (1953) has done in his classic experiment consisting in the demonstration of the formation of amino acids by electric discharges in a gaseous mixture of hydrogen, carbon dioxide, ammonia and water. His next paper (Miller 1955) presented the possibility of formation of more complicated compounds, including polymers. One can conclude that all sources of energy able to start formation of reactive species are potentially friendly to the origins of life, also, possibly in other places of the Universe.

The Early Earth was from the beginnings penetrated by ionizing radiation, of intensity much higher than now. The origins of radiations were very different, from sources present on the Earth, like radiations of radioactive elements, to radiations coming from outer space like cosmic radiation. Therefore all kinds of ionizing radiations were represented, of different particles and quanta and of very different quality expressed by their LET value (linear energy transfer) (Zagórski 2010a, b, c). The chemical action of ionizing radiation is more “diluted” (calculating it to the unit of volume) in comparison to Miller’s experiment using electric discharges in gaseous mixtures of compounds of carbon, hydrogen, oxygen, nitrogen and sulphur and therefore was not more closely investigated. Future investigation cannot be limited to the gas phase but should be extended to aqueous systems (including the Darwinian “small pond”), containing radioactive potassium and multiphase systems, e.g. uranium ore immersed in aqueous solutions of proper starting compounds. Anyway, sources of energy as “software” can work creatively only in suitable locations, in analogy to “hardware” in computer calculations (Zagórski 2010a).

One can expect similar products of ionizing radiation interaction as with electric discharges and the same main trouble, i.e. production of racemic amino acids, without any enantiomorphic excess. Chemical changes induced in the media by radiation are of prebiotic character but could not alone be responsible for the decisive (as far we know) character for the formation of life. For instance they could not contribute to the separation of racemic mixtures into separate enantiomorphic species. In spite of no optical activity segregation, one can call ionizing radiation and its cousins in the high energy chemistry family friends to the origins of life chemistry. That field of research is not exhausted yet and many prebiotic or probiotic reactions hopefully will be found with active involvement of ionizing radiation in the formation of different organics.

Coming to the second face of ionizing radiation connections to life, are chemical effects connected with modification of the molecules of life. They can be of destructive character but sometimes play a supporting role by positive action in biological evolution. Omnipresent ionizing radiation was acting on every sort of chemical compounds in the chain of origin of life and evolution of the biosphere, from prebiotic compounds, sometimes created with the participation of ionizing radiation to more or less developed organisms classified as living creatures. The action of radiation can be a direct one on molecules absorbing it, or an indirect one, by products of radiolysis of the medium on dispersed compounds in it and on organisms. Even high LET value radiations of low penetration, like alphas from radon, abundant on early Earth, were of enormous influence, because they were able to penetrate everything exposed to the air, including the first living creatures inhaling the air (Zagórski 2010b). Whatever the detailed chemical effects, investigated and generalized by principles of radiation chemistry, absorption of ionizing radiation means a supply of energy to the system, participating in the so called “chemical evolution” (no direct analogy to the Darwinian biological evolution). Chemical changes induced in the media by radiation were of prebiotic character but could not alone be responsible for decisive (as far we know) character for the formation of life. For instance, as mentioned before, they could not contribute to the separation of racemic mixtures into separate enantiomorphic species.

Organics in meteorites, whatever the mechanisms of the formation from carbon, are exposed to different ionizing radiations during travel in space and in effect, are modified. For instance, the Cataldo group is devoted in a series of publications (Cataldo et al. 2011a, b, c) to investigation of the radiolysis of amino acids, known from their presence in meteorites. Radiation induced changes of organic compounds start from dehydrogenation (Zagórski 2006a, b)—energetically the easiest way; later comes deamination and decarboxylation. These phenomena exclude a possibility of transfer of life from far corners of the Universe, the concept still alive as the panspermia hypothesis (Zagórski 2007).

Answering the question in the title of the summary, one can say that the ionizing radiation could be a “friend” as being involved in creation of organics (e.g of methane from carbon dioxide, Zagórski et al. unpublished), or polymerization of acetylene, probably present in aqueous systems near volcanos).

As concerns radiation being a “foe”, one can consider the depolymerization action on compounds already formed before. On the other hand, the chemical bond’s disruptive action on information transmitting compounds (RNA and later DNA) was contributing to mutations, decisive elements in the Darwinian evolution of Life. In conclusion, the role of ionizing radiation in origins of life and early evolution cannot be neglected and demands further research in both categories of friend and foe.
